# Evaluation of Additional *Drosophila suzukii* Male-Only Strains Generated Through Remobilization of an FL19 Transgene

**DOI:** 10.3389/fbioe.2022.829620

**Published:** 2022-03-15

**Authors:** Akihiko Yamamoto, Amarish K. Yadav, Maxwell J. Scott

**Affiliations:** Department of Entomology and Plant Pathology, North Carolina State University, Raleigh, NC, United States

**Keywords:** *Drosophila* suzukii, piggyBac transposon, sterile insect technique, spotted wing drosophila, fsRIDL

## Abstract

*Drosophila suzukii (D. suzukii)* (Matsumura, 1931; Diptera: Drosophilidae), also known as spotted wing *Drosophila*, is a worldwide pest of fruits with soft skins such as blueberries and cherries. Originally from Asia, *D. suzukii* is now present in the Americas and Europe and has become a significant economic pest. Growers largely rely on insecticides for the control of *D. suzukii*. Genetic strategies offer a species-specific environmentally friendly way for suppression of *D. suzukii* populations. We previously developed a transgenic strain of *D. suzukii* that produced only males on a diet that did not contain tetracycline. The strain carried a single copy of the FL19 construct on chromosome 3. Repeated releases of an excess of FL19 males led to suppression of *D. suzukii* populations in laboratory cage trials. Females died as a consequence of overexpression of the tetracycline transactivator (tTA) and tTA-activated expression of the *head involution defective* proapoptotic gene. The aim of this study was to generate additional male-only strains that carried two copies of the FL19 transgene through crossing the original line with a *piggyBac* jumpstarter strain. Males that carried either two chromosome 3 or a singleX-linked transgene were identified through stronger expression of the red fluorescent protein marker gene. The brighter fluorescence of the X-linked lines was likely due to dosage compensation of the red fluorescent protein gene. In total, four X-linked lines and eleven lines with two copies on chromosome 3 were obtained, of which five were further examined. All but one of the strains produced only males on a diet without tetracycline. When crossed with wild type virgin females, all of the five two copy autosomal strains examined produced only males. However, the single copy X-linked lines did not show dominant female lethality. Five of the autosomal lines were further evaluated for productivity (egg to adult) and male competition. Based on these results, the most promising lines have been selected for future population suppression experiments with strains from different geographical locations.

## Introduction

First reported in 2008 in California and Europe, *Drosophila suzukii (D. suzukii)* is now widely found through North America, Europe and some locations in South America (Cini et al., 2014; Asplen et al., 2015; Tait et al., 2021). Unlike most *Drosophila* species that are not economic pests, *D. suzukii* females lay their eggs in ripe fruit before harvest (Hauser, 2011). The species is commonly known as spotted wing *Drosophila* since adult males have a dark spot that is clearly seen on each wing (Hauser, 2011). Growers largely rely on insecticides for control but use is weather-dependent and resistance to the chemicals is anticipated as seen with Spinosad in California (Gress and Zalom, 2019). *D. suzukii* have a wide range of non-crop host plants, which can serve as a refuge (Lee et al., 2015; Kenis et al., 2016). Thus, reinfestation of crops following insecticide treatment can be relatively rapid (Tait et al., 2021). Additional area-wide control methods are clearly needed.

One promising approach for area-wide control of insects is the release of fertile males carrying dominant female lethal genes (Heinrich and Scott, 2000; Thomas et al., 2000), which is also known as fsRIDL (female-specific release of insects carrying a dominant lethal genetic system) (Alphey, 2014). Wild type virgin females that mate with released fertile fsRIDL males will only produce male offspring. Modeling indicates that repeated releases of an excess of fsRIDL males can lead to suppression of pest populations (Schliekelman and Gould, 2000; Thomas et al., 2000). The fsRIDL strains can be reared in the laboratory or a mass-rearing facility as a conditional system is used for controlling expression of the female-specific lethal gene. Conditional expression is achieved by using the tetracycline transactivator (tTA), a transcription factor that binds very specifically to a sequence from the *Escherichia coli* tet operator (tetO) (Gossen and Bujard, 1992). The binding of tTA to the tetO is inhibited by adding tetracycline to the diet, thus providing a simple off-switch. In the initial system we developed (Heinrich and Scott, 2000), the lethal or effector gene cassette consisted of seven copies of tetO, a core promoter and the coding sequence for the *head involution defective* (*hid*) proapoptotic gene. Widespread expression of *hid* in *D. melanogaster* led to organismal death (Grether et al., 1995). Female-specificity was achieved by using an enhancer-promoter from a yolk protein gene to drive tTA expression (Heinrich and Scott, 2000). Subsequently, fsRIDL strains were simplified to a single component system that consisted of a tTA activated enhancer-promoter driving expression of tTA (Fu et al., 2007; Ant et al., 2012). In this autoregulatory system, very high levels of tTA gene expression led to organismal death likely due to “transcriptional squelching” or inhibition of ubiquitin-mediated proteolysis (Fu et al., 2007). Only females died on a diet without tetracycline as the tTA coding region was interrupted by the sex-specifically spliced first intron from the *Ceratitis capitata transformer* (*tra*) gene (Fu et al., 2007). Similarly, the initial New World screwworm (*Cochliomyia hominivorax*) fsRIDL strains carried single component tTA overexpression transgenes but with the sex-specific intron from the *C. hominivorax tra* gene (Concha et al., 2016). The female-specific tTA overexpression systems were functional in *D. melanogaster*, indicating that the screwworm and *C. capitata tra* introns were correctly spliced in *D. melanogaster* (Fu et al., 2007; Li et al., 2014). We recently developed the FL19 *D. suzukii* fsRIDL strain that had a female-specific tTA overexpression gene and a tTA activated *hid* gene in a single construct (Li et al., 2021).

The effectiveness of fsRIDL strains for population suppression has been demonstrated in cage trials. In the continuous population experiments, conditions were first established for maintaining a population simply by providing sufficient diet. Subsequently, repeated releases of an excess of fertile fsRIDL males led to eradication of the populations (Ant et al., 2012; Leftwich et al., 2014; Harvey-Samuel et al., 2015). With *D. suzukii*, repeated releases of FL19 males (approximately 10–13:1 ratio) led to a sharp reduction in egg production in the first month and by 8 weeks the test cages had stopped laying eggs (Li et al., 2021). Males from a fsRIDL strain of the diamondback moth, *Plutella xylostella*, have been tested in cages (Harvey-Samuel et al., 2015) and in the open field (Shelton et al., 2020). For the latter, the fsRIDL males showed excellent dispersal and persistence (Shelton et al., 2020).

In the field, fsRIDL males will likely encounter females from populations with much greater genetic diversity than found in lab strains (Lewald et al., 2021). To investigate the sensitivity of a female-specific tTA overexpression system to variation in genetic background, we utilized the *D. melanogaster* Genetic Reference Panel (DGRP) that consists of 205 highly inbred lines each with fully sequenced genomes (Mackay et al., 2012). Males from an fsRIDL strain were crossed with virgin females from each DGRP line and the number of male and female offspring counted. The level of female lethality between DGRP lines varied considerably from 11% to 97% with a broad sense heritability of 0.89 (Knudsen et al., 2020). We concluded that genetic background could have a significant impact on the efficacy of the tTA overexpression system. This was one reason why a second effector, *hid*, was included in the FL19 construct. The aim of this study was to develop robust *D. suzukii* fsRIDL strains that either carried two copies of the FL19 transgene or carried the FL19 transgene at a favorable chromosomal location as the tTA expression system is sensitive to position-effects (Heinrich and Scott, 2000; Horn and Wimmer, 2003). This was achieved by remobilizing the original FL19 transgene through crossing with a *piggyBac* jumpstarter strain that expresses *piggyBac* transposase in the germline (Chu et al., 2018). Here we report on the new *D. suzukii* fsRIDL strains obtained by using this approach.

## Materials and Methods

### Fly Rearing, Transposition and Recombination Mapping

**FIGURE 1:**
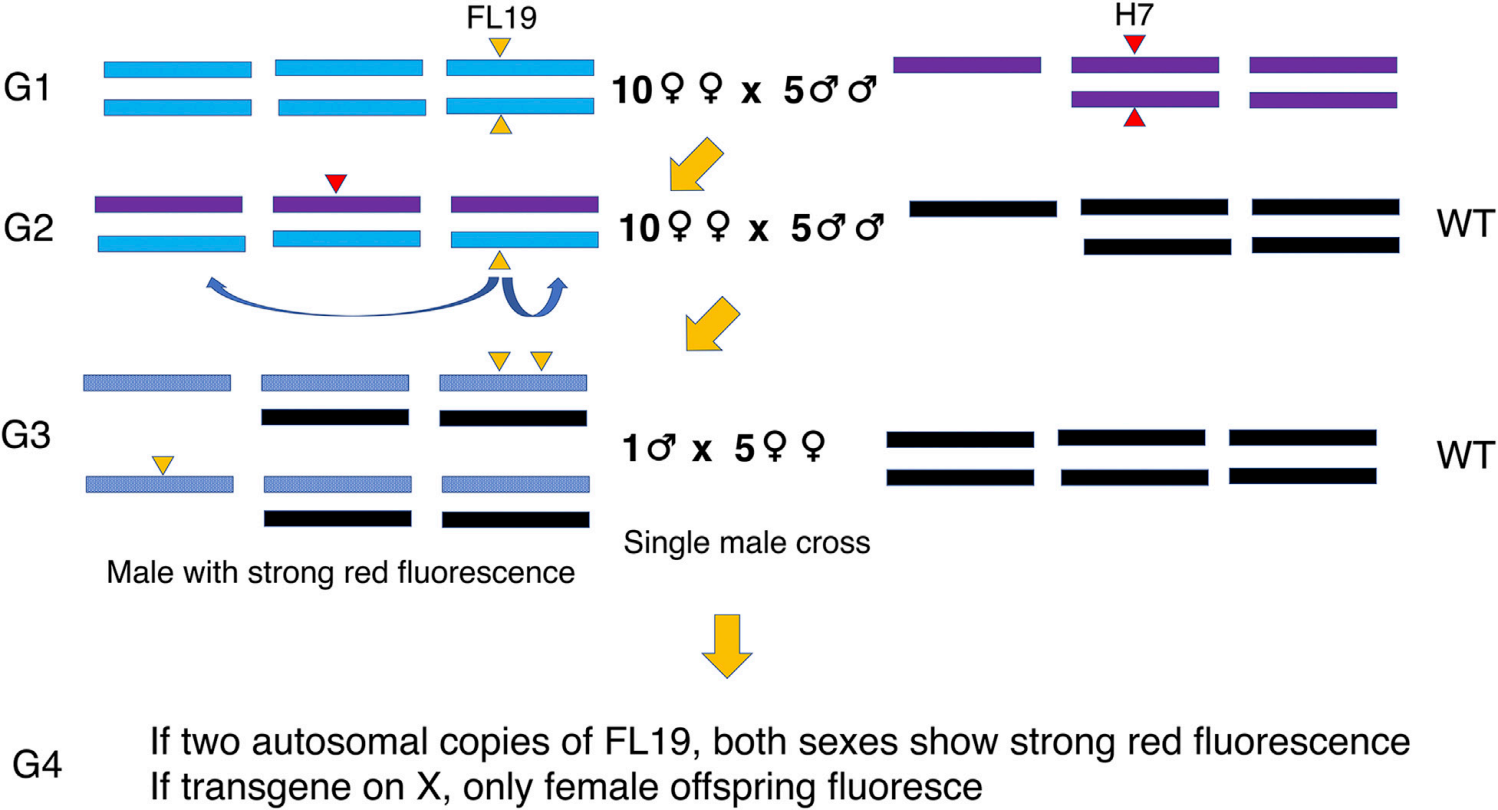
Mating scheme to produce and identify males with a transposed FL19 transgene. FL19 females were crossed with the H7 *piggyBac* transposase jumpstarter males to initiate transposition. Males that could be carrying an X-linked or two autosomal copies of FL19 were identified by fluorescence intensity in G3.

*D. suzukii* were raised on cornmeal-yeast-agar diet at room temperature (approx. 20–22°C) in the open laboratory. The relative humidity in the lab was between 20% and 50% and the lights were on for about 12 h on most days. The original wild-type colony was established from infested fruit collected from a field in North Carolina in 2011 (Burrack et al., 2013) and the initial FL19 strain was previously made by *piggyBac*-mediated germline transformation of this wild type strain (Li et al., 2021). The wild type colony was periodically genetically augmented with flies collected in North Carolina (Elsensohn et al., 2021). The newly refreshed and the original 2011wild type colonies are maintained separately by our lab. To remobilize the FL19 transgene, ten FL19 virgin females were crossed with five males from the H7 *piggyBac* jumpstarter strain (Chu et al., 2018) (Figure 1). From the offspring of the cross, ten virgin females were crossed to five wild type males. The male offspring were screened for bright red fluorescence using a M205FA microscope (Leica Microsystems, Buffalo Grove, IL) with the DsRed filter [ex 545/25, em 595/50 nm]. Individual candidate males were then each crossed with five wild type virgin females. If the FL19 transgene had transposed to the X chromosome, then only the female offspring would show red fluorescence. If both sexes showed bright red fluorescence, this could indicate that the flies carried two autosomal copies of FL19. Homozygous lines for each putative transposition event were established by crossing and selecting for particularly high levels of red fluorescence.

For recombination mapping of X-linked FL19 transgenes, six crosses were set for each combination FL19(X) lines. The double heterozygous virgin female offspring were collected and crossed with wild type males. Recombinant male offspring that showed no red fluorescence was identified and counted. A map distance of the two FL19(X) sites were calculated by; map distance (cM) = 100 x (2 x number of non-fluorescent males)/total males.

### Assessment of Female-specific Lethality

To assess the level of female lethality in the homozygous strains, three vials were set on the same day with five pairs on a diet that either contained tetracycline (40 μg/ml) or lacked tetracycline. Parents were transferred three times to new cultures every 3 days to create four cultures. To determine if the strains showed dominant female lethality, five transgenic males were crossed with five wild type virgin females. Parents were transferred to new cultures every 3 days to create four cultures. Two replicates were set each on tetracycline and non-tetracycline foods. The number of male and female offspring from each cross were counted daily until 20 days after setting the cross.

### Assessment of Strain Productivity

Cut vials, which were regular fly vials cut at the middle to create 7.2 cm-long tubes and 2.3 cm-deep cups, were used for easy handling of eggs. A tube and a cup were taped together to create a vial for productivity tests. The cup contained about 10 ml of culture medium. Flies that were raised under non-crowded conditions were collected from several vials 3 to 14 days after eclosion and then allowed to lay eggs in the cut vials for about 20 h. The cut vials were then separated, eggs were picked with a needle and transferred to a new vial with about 10 ml of medium. Typically, 50 eggs were transferred to each vial. After 2 days unhatched eggs were counted. Emerging adults were sexed and counted up to 3 weeks after the egg-picking. At the end of emergence of adult flies, the number of pupal cases was counted. The egg survival ratio is the number of hatched eggs divided by the total number of eggs. The larval survival ratio is the number of pupae divided by the number of hatched eggs. The pupal survival ratio is the number of adults divided by the number of pupae. The egg to adult survival ratio is the number of adults divided by the total number of eggs. This ratio was multiplied by two for the transgenic lines on diet without tetracycline.

### Male Mating Competitiveness

Ten transgenic males from strains reared without tetracycline in the diet and 10 wild type males were introduced into an 8 oz bottle with diet and left undisrupted for 1 hour. Ten wild type virgin females were added to the bottle which was kept undisrupted at room temperature (∼22°C) for approximately 24 h. All flies were four to 6 days old. Females were transferred individually to fresh vials with diet. The offspring of each female were counted, sexed and examined for fluorescence status (presence/absence) to determine whether the female had mated with an FL19(3 + 3) or wild type male. The presence of both fluorescent and non-fluorescent offspring would indicate remating during the 24 h period when males were with females. Four to eight bottles were set for each line. Mate success ratio of each line was calculated by; success ratio = (number of females with fluorescent sons only + a half number of females with both fluorescent and non-fluorescent sons)/number of fertile females.

### Molecular Analysis

The genomic location of the transgenes was determined using inverse PCR with primers for the *piggyBac* left and right ends as previously described (Li et al., 2001 and see Supplementary Table S1). If inverse PCR was only successful for one end, confirmation of the transgene location was obtained by PCR using one primer for the *piggyBac* end and one primer based on the flanking genome sequence of the insertion site.

### Statistical Analysis

For the female lethality tests (Table 4), contingency analysis (fit Y by X) was performed using JMP Pro 15 (SAS Institute). The egg to adult ratio (Table 5) was analyzed in SAS (Version 9.4, Cary, NC) on a diet with tetracycline, with the total number of adults divided by the number of eggs as the response variable in PROC LOGISTIC, and both line, tetracycline (+/-), and their interaction as predictor variables. All effects were statistically significant (*p* < 0.0001). For diet without tetracycline, the number of male eggs was estimated as the floor of the number of eggs divided by two. Least-squares means were obtained for each treatment combination, and the specific differences of interest were calculated. The log-odds ratio of the two treatments (each line compared to each wild type for both tetracycline + and tetracycline -) were obtained. To control for multiple tests, a Bonferroni correction was used where the adjusted *p*-value to determine statistical significance was set at 0.05/30 = 0.0016 for a diet with tetracycline and 0.05/15 = 0.0033 for a diet without tetracycline (only males produced from transgenic lines). The male competitiveness data (Table 6) were also analyzed in SAS, Version 9.4 (Cary, NC). The mating competitiveness index (MCI) was recalculated as the ceiling of the number mated with transgenic plus half the number that remated divided by the total number mated. This allowed the count to remain an integer. A z-test for one proportion was run for each line comparing the MCI to the null hypothesized proportion of 0.5.

## Results

### Transposition of the FL19 Transgene to New Chromosomal Locations

The original FL19 male-only strain was made by *piggyBac* transposase-mediated germline transformation (Li et al., 2021). The transgene was located on chromosome 3 between the *DsShal* and *DsCG9231* genes. The *piggyBac* H7 jumpstarter strain efficiently mediates remobilization of *piggyBac* transgenes (Chu et al., 2018). To remobilize the FL19 transgene, FL19 virgin females were crossed with H7 males and virgin female offspring collected (Figure 1). As X-linked transgenes are generally dosage compensated in male *Drosophila melanogaster* (Spradling and Rubin, 1983; Fitzsimons et al., 1999), we reasoned that G3 males carrying a single X-linked FL19 transgene would show an increased expression of the red fluorescent protein marker gene (Figure 1). Males that carried FL19 at the original location and at a second autosomal location would also show brighter red fluorescence. Such transpositions tend to be predominately local (Daniels and Chovnick, 1993), so the expectation was that males would carry FL19 at the original location and at a closely linked site on chromosome 3. X-linked transposition events were identified through crossing putative males with wild type virgin females (Figure 1). From approximately 25,000 G3 males, we derived four X-linked lines and eleven lines with two copies of FL19 on chromosome 3 (Table 1). Of the third chromosome lines, one was homozygous lethal and two lines were homozygous sterile (Table 1). Of the remaining eight homozygous viable lines, five (8, 36, 40, 70 and 75) that were vigorous on diet with tetracycline were selected for further study.

**TABLE 1:** X-linked and third chromosome FL19 lines.

Chromosome	Line	Homozygous condition
X	7	viable
X	46	viable
X	77	viable
X	79	viable
3	F8	sterile
3	6	sterile
3	8	viable
3	17	viable
3	18	dead
3	36	viable
3	40	viable
3	70	viable
3	75	viable
3	78	viable
3	83	viable

**FIGURE 2:**
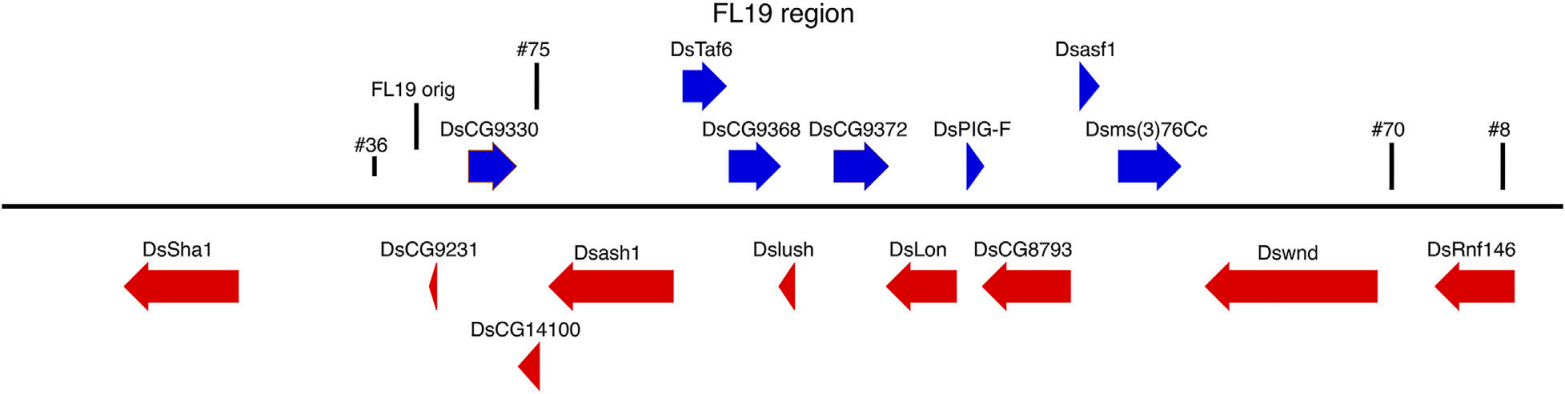
Relative location of FL19 transgenes on chromosome 3. The location of transgenes is indicated by vertical lines. The length and direction of arrows indicates the sizes and direction of transcription of transcription of genes as annotated (Paris et al., 2020).

The chromosomal locations of the X-linked and most of the chromosome 3 lines were determined by inverse PCR and blast searches of the assembled *D. suzukii* genomes (Chiu et al., 2013; Paris et al., 2020) (Table 2). Some locations could not be determined as the transgene appeared to be located within a repetitive sequence. PCR analysis confirmed that all the chromosome 3 lines carried two copies of FL19, with one copy at the original location near the *DsCG9231* gene (Figure 2). The other copies of FL19 were found to have inserted no more than 68 kb from the original location (Table 5; Figure 2). In line 36, the additional FL19 transgene is less than 3 kb from the original and is also within the intergenic region between the *DsCG9231* and *DsSha1* genes (Figure 2). In line 70, the additional FL19 transgene is also in an intergenic region, between the *Dswnd* and *DsRnf146* genes (Table 2; Figure 2). In the other lines, 8 and 75, the transgene is located within genes. In line 8, the transgene is within an intron of the *DsRnf146* gene. In the line 75 the transgene is within an exon of the *DsCG14100* gene and would likely disrupt gene function.

**TABLE 2:** FL19 insertion sites in transposition lines.

Line	Chromosome	Insertion site sequence (TTAA in bold)	Nearest gene	Relationship to original FL19 location
7	X	TCG​ATA​TCA​GGT​GGT​GCA​CT﻿**TTA​A**GG​AGT​TGG​AGC​ATA​GCA​TAT	*DsCG8661* (contig 7^a^)	NA
46	X	GCT​CCG​CCG​TCG​TTT​GTA​TT**T​TAA** TTT​AGC​CTC​TTC​AAA​TTG​CT	*DsCG32655* (>20 kb) (contig 11)	NA
77	X	CGC​CAA​AAC​GCA​AGA​AAC​CT**T​TAA**​AAG​AGT​AAT​CCA​GAT​AAT​GG	*DsCp110* (contig 15)	NA
79	X	ND (repetitive)	ND	NA
8	3	TAA​ATA​ATT​TCG​AAA​CCA​CT**T​TAA**​AAA​GAA​CTT​TGT​AGT​TTA​GT	*DsRnf146* (intron)	67.8 kb 3′
36	3	﻿TTG​TAA​ATT​AAA​ATA​AAG​GC**T​TAA**​CTA​AAA​AAA​GTA​CCA​AGA​AC	*DsCG9231*	2.6 kb 5′
40	3	ND	ND	ND
70	3	GAG​GAT​CAT​GTT​GAT​GCC​CA**T​TAA**​ACC​GGC​CAA​GCT​CAG​AAG​CA	*Dswnd* and *DsRnf146*	60.8 kb 3′
75	3	CGT​GTT​TAC​CGG​TTC​GTG​C**TT​AA**A​CTT​GAA​TTC​CCG​AAG​AGA​T	*DsCG14100* (coding)	7.5 kb 3′

a Contigs of the Paris et al. (2020) genome assembly.

In the X-linked lines, three of the four FL19 transgenes appear to be found at widely separated locations as their flanking sequences each align to a different contig of the genome assembly (Paris et al., 2020) (Table 2). The location of the fourth transgene (#79) could not be determined by inverse PCR. In line 46 the transgene is in a gene-poor region of the X chromosome with the nearest genes more than 20 kb distant. We next used recombination mapping to determine the genetic distance between each of the transgenes. The transgene in line 46 was found to be 45 cM from the transgene in line 79 (Table 3). The transgenes in lines 77 and 79 appear to be very closely linked locations as we did not recover any recombinants (Table 3). Lastly, in line 7 the transgene mapped to a region between the 46 and 79 transgenes (Table 3).

**TABLE 3:** Recombination mapping of X-linked FL19 transgenes.

Cross	Number fluorescent F_1_ males	Number wild type F_1_ males	Map distance (cM)
79♀ x 46♂	114	33	45
46♀ x 7♂	99	18	31
79♀ x 7♂	151	9	11
77♀ x 79♂	136	0	0
7♀ x 77♂	114	5	8

### Tetracycline-Repressible Female Lethality

All the lines can be readily maintained on diet supplemented with tetracycline. When raised on diet that lacked tetracycline all lines produced 99–100% males except for the X-linked line 46, which gave 73.5% males (Table 1). In a future field release, flies would be raised on diet without tetracycline and the released fertile males would mate with wild type females. Ideally, all the female offspring would die. Therefore, we next collected males from the lines raised on diet without tetracycline and crossed to wild type virgin females. For the chromosome 3 lines that carry two copies of the FL19 transgene, all lines showed dominant female lethality (Table 4). However, none of the X-linked single copy lines showed dominant female lethality. Two lines, 7 and 77, produced significantly more male than female offspring (Pearson’s Chi-squared test, *p* < 0.0001). On a diet with tetracycline, approximately an equal number of males and females were produced from the crosses of transgenic males with wild type females.

**TABLE 4:** Tetracycline-repressible female-specific lethality of FL19 transposition lines.

Strain (chromo-some)	Tetra-cycline	Homozygous Number Males^a^	Homozygous Number Females	Homoyzgous %Male	Hemizygous Number Males	Hemizygous Number Females	Hemizygous % Males
7 (X)	−	239	0^c^	100	239	81^c^	74.7
	+	253	221	53.3	294	286	50.7
46 (X)	−	119	43^c^	73.5	186	229^d^	44.8
	+	188	234	44.5	192	206	48.2
77 (X)	−	268	0^c^	100	236	52^c^	82
	+	283	243	53.8	264	275	49
79 (X)	−	261	1^c^	99.6	226	274^d^	54.8
	+	188	242	43.7	274	289	48.7
7 (X) + FL19	−	66	0^c^	100	115	0^c^	100
	+	54	42	56.2	146	166	46.8
8 (3)	−	60	0^c^	100	179	0^c^	100
	+	46	55	45.5	263	258	50.5
36 (3)	−	64	0^c^	100	245	0^c^	100
	+	61	32	65.6	198	248	44
40 (3)	−	34	0^b^	100	51	0^c^	100
	+	74	34	68.5	139	161	46.3
70 (3)	−	40	0^b^	100	190	0^c^	100
	+	89	29	75.4	258	225	53.4
75 (3)	−	20	0^b^	100	176	0^c^	100
	+	49	21	70	257	290	53.8

a Total count of offspring from three independent vials of flies except for the homozygous chromosome 3 and X + FL19 lines where the data is from the productivity experiment shown in Table 1.

b The number of females obtained was significantly lower than expected (Pearson’s Chi-squared test, *p* < 0.001).

c The number of females obtained was significantly lower than expected (Pearson’s Chi-squared test, *p* < 0.0001).

d The number of females obtained was not significantly lower than expected.

### General Fitness and Male Sexual Competitiveness of Transgenic Sexing Strains

One of the fitness measurements that is important in a mass rearing facility is the percentage of eggs that produce adults (Concha et al., 2016; Concha et al., 2020). We firstly measured the proportion of eggs that produced adults in our two wild type North Carolina colonies. One colony has been periodically refreshed with flies collected in North Carolina and the other has been continuously maintained in the laboratory since 2011. The 2011 wild type strain showed significantly reduced adult production compared to the newly refreshed strain on diet with or without tetracycline (*p* < 0.0001) (Table 5). Similarly, on diet with tetracycline, the adult production for all of the transgenic lines was significantly less than the newly refreshed wild type strain (Table 5). However, since the original FL19 line was by injecting embryos from the 2011 wild type strain, we also compared each of the transgenic lines to that strain. Compared to the 2011 wild type strain, adult production on a diet with tetracycline and male production on a diet without tetracycline was not significantly different in five of the seven lines (FL19, 8, 36, 40, 7(X) + FL19). Two of the transgenic lines, 70 and 75, did show significantly reduced adult production on both diets compared to the 2011 wild type strain (Table 5). Several of the transgenic lines (e.g., 8, 40, 70) showed a wider variation between replicates for pupal and adult survival on a diet without tetracycline than on a diet with tetracycline (see standard deviation (SD) values in Table 5). This variability in male survival suggests there may be some leaky expression of the lethal genes, particularly in some lines.

**TABLE 5:** Productivity of FL19 transposition lines.

Strain (chromosome)	Tetra-cycline	Number eggs^a^	Number unhatched eggs	Number pupae	Numbermales	Number females	Number total adults	Egg survival ratio Mean (SD)	Larval survival ratio Mean (SD)	Pupal survival ratio Mean (SD)	Egg to adult ratio^b^ Mean (SD)
Wild type	**−**	150	10	133	65	59	124	0.93 (0.046)	0.95 (0.111)	0.93 (0.033)	0.83 (0.11)
	**+**	183	15	165	67	89	156	0.92 (0.023)	0.97 (0.1)	0.95 (0.051)	0.85 (0.103)
Wild type (est. 2011)	**−**	230	30	157	69	70	139	0.87 (0.064)	0.79 (0.1)	0.89 (0.076)	0.62** (0.121)
	**+**	200	20	117	43	62	105	0.84 (0.054)	0.70 (0.082)	0.90 0.11)	0.53** (0.079)
7 (X) + FL19 (3)	**−**	200	16	70	66	0	66	0.92 (0.016)	0.38 (0.074)	0.94 (0.011)	0.66^NS^ (0.12)
	**+**	200	14	112	54	42	96	0.93 (0.035)	0.6 (0.178)	0.87 (0.091)	0.48^NS^ (0.099)
FL19 (3)	**−**	600	63	186	157	0	157	0.89 (0.041)	0.35 (0.1)	0.86 (0.11)	0.52^NS^ (0.145)
	**+**	600	70	350	161	150	311	0.88 (0.05)	0.66 (0.106)	0.89 (0.073)	0.52^NS^ (0.08)
8 (3)	**−**	282	18	83	60	0	60	0.94 (0.049)	0.32 (0.13)	0.66 (0.3)	0.44^NS^ (0.32)
	**+**	200	16	112	46	55	101	0.92 (0.051)	0.61 (0.13)	0.9 (0.06)	0.51^NS^ (0.117)
36 (3)	**−**	281	70	64	54	0	54	0.75 (0.071)	0.3 (0.13)	0.87 (0.14)	0.37^NS^ (0.135)
	**+**	146	36	61	27	32	59	0.75 (0.05)	0.55 (0.06)	0.97 (0.03)	0.40^NS^ (0.053)
40 (3)	**−**	142	50	34	22	0	22	0.65 (0.08)	0.37 (0.09)	0.60 (0.42)	0.3^NS^ (0.27)
	**+**	166	53	74	36	34	70	0.68 (0.023)	0.66 (0.113)	0.96 (0.057)	0.42^NS^ (0.051)
70 (3)	**−**	200	48	40	23	0	23	0.76 (0.059)	0.26 (0.088)	0.53 (0.33)	0.23* (0.21)
	**+**	236	69	89	56	29	85	0.71 (0.069)	0.53 (0.083)	0.96 (0.057)	0.36* (0.071)
75 (3)	**−**	217	90	20	10	0	10	0.59 (0.102)	0.17 (0.117)	0.43 (0.159)	0.1* (0.092)
	**+**	200	79	48	25	21	46	0.6 (0.066)	0.4 (0.047)	0.94 (0.04)	0.23* (0.038)

a Total count of offspring from at least three replicates.

b On diet without tetracycline this is the number of males divided by the number of eggs times two. On diet with tetracycline this is the total number of adults divided by the number of eggs. NS, indicates not significantly different compared to wild type (est. 2011). * indicates significantly reduced adult production compared to wild type (est. 2011) (*p* < 0.0016 for + tetracycline and *p* < 0.0033 on no tetracycline, see methods for details). ** indicates significantly reduced adult production compared to wild type (newly established).

The sexual competitiveness of the males from the lines was assessed by presenting virgin wild type females with equal numbers of transgenic and wild type males as done previously with the *C. hominivorax* male-only strains (Concha et al., 2016). As previously, the MCI was calculated where an index of 0.5 indicates the transgenic and wild type males are equally competitive. Males from the original FL19 strain and from most of the new male-only lines were significantly out-competed by the wild type males (Table 6). However, males from line 36 and the combined 7 (X-linked) with the original FL19 strain competed effectively with the wild type males, with MCI values not significantly different than the expectation of 0.5 if fully competitive (Table 6).

**TABLE 6:** Male sexual competitiveness.

Line (chromosome)	Number replicates	Number mated with transgenic	Number mated with wild type	Number mated with both males (remating)	Total mated	MCI^a^ (SE)	*p*-value
FL19 (3)	7	16	48	3	67	0.27 (0.06)	0.0002
7 (X) + FL19 (3)	4	14	23	1	38	0.39 (0.08)	0.1944
8 (3)	6	16	40	0	56	0.29 (0.07)	0.0013
36 (3)	5	25	18	1	44	0.59 (0.08)	0.2278
40 (3)	8	15	46	9	70	0.29 (0.06)	0.0003
70 (3)	7	14	46	3	63	0.25 (0.06)	<0.0001
75 (3)	4	7	26	1	34	0.24 (0.09)	0.0020

a Mating competitiveness index or MCI, is the number mated with transgenic plus half the number that remated divided by the total number mated.

## Discussion

In this study, the FL19 transgene reported recently (Li et al., 2021) was remobilized to new locations on the third and X chromosomes. As expected, the third chromosome jumps were relatively short with the most distant transgene less than 70 kb from the original FL19 location. All chromosome 3 lines had two transgenes, with one at the original location. This could occur by transposition to the non-donor sister chromatid during meiosis after DNA replication as seen with *P* element transposition (Daniels and Chovnick, 1993). Alternatively, transposition could occur to the donor sister chromatid followed by repair using the non-donor sister chromatid as template. All chromosome 3 lines showed 100% dominant female lethality when transgenic males were crossed with wild type females on diet without tetracycline. The most promising of the five lines examined are 8, 36 and 40 as the level of production of adults from eggs was comparable to the original wild type strain. Of these, line 36 males were sexually competitive with wild type males. Males from all other chromosome 3 lines, including the original FL19 strain, were significantly less competitive than wild type males. It is not obvious why line 36 males would be more competitive than males from the original FL19 line. Perhaps by chance, changes in the genetic background produced a strain with improved male competitiveness. If so, it may be beneficial to backcross transgenic males with females from the recently refreshed wild type strain, which showed significantly higher productivity compared to the wild type strain that has been in the lab for about 10 years. While none of the X-linked lines showed dominant female lethality, two lines, 7 and 77, produced mostly males. Further, 100% of homozygous females died when reared on diet without tetracycline. While not desirable for fsRIDL, these lines could be considered for a sterile release program with the males sterilized by exposure to radiation (Sassu et al., 2019). The level of dominant female lethality could be increased by making a recombinant strain that carries two transgenes, one each from the 7 and 77 lines. However, this could be challenging without balancer chromosomes. If female lethality is fully dominant, matings with wild type females would produce only males that would not carry any transgenes. This could be desirable if there is concern about transgene persistence in the field (Evans et al., 2019). Since line 7 showed a high level of female lethality, we combined it with the original FL19 line and bred to homozygosity for both transgenes. This strain, 7 (X) + FL19, appears to be quite promising as productivity was comparable to the long established wild type strain and males were sexually competitive with wild type males. A disadvantage of this strain for an fsRIDL program is that the male offspring from matings with wild type females would only inherit the chromosome 3 transgene.

The frequency of remobilization seen in this study was much lower than reported previously with the H7 *piggyBac* jumpstarter strain (Chu et al., 2018). This was most likely because our screen would have missed simple cut and paste transposition events to autosomal locations as most would not have produced males with significantly increased expression of the red fluorescent protein gene. In contrast, Chu et al., 2018 used donor strains that carried a fluorescent protein gene that was sensitive to position-effects, likely nearby transcription enhancers. Consequently, transposition events could be detected by changes in the expression pattern of the fluorescent protein gene.

One aim of this study was to produce strains with two copies of the FL19 transgene that could be tested against strains with different genetic backgrounds. For example, by crossing transgenic males with virgin female wild type flies from Western and Eastern US populations which are genetically quite distinct (Lewald et al., 2021). Several strains made in this study (8, 36, 40, 7(X)+FL19) would be worth evaluating since they show high female lethality and productivity comparable to the older wild type strain. For fsRIDL, modeling has shown that it would be advantageous if each autosome (i.e. chromosomes 2, 3 and 4) carried a copy of the dominant female lethal transgene (Schliekelman and Gould, 2000; Thomas et al., 2000). This could be accomplished by targeting FL19 to specific chromosomal locations using CRISPR/Cas9. For this approach the FL19 gene construct and fluorescent protein gene would be flanked with homologous sequences from the region targeted rather than the 5′ and 3’ ends of the *piggyBac* transposon as used in this study. Cas9-mediated cleavage of genomic DNA followed by homology-dependent repair using the injected plasmid DNA as template should lead to integration of the FL19 construct at the targeted location (Wang et al., 2015; Davis et al., 2018). These experiments would be facilitated by using the lines that express Cas9 in the germline that we described recently (Kandul et al., 2021).

## Data Availability

The original contributions presented in the study are included in the article/Supplementary Material, further inquiries can be directed to the corresponding author.
