# Supplementary material for: Evaluation of Additional Drosophila suzukii Male-Only Strains Generated Through Remobilization of an FL19 Transgene
Source: Front Bioeng Biotechnol. 2022 Mar 15;10:829620. doi: 10.3389/fbioe.2022.829620 (PMC8965018; doi:10.3389/fbioe.2022.829620)
Supplement: Supplementary file 1 [file Table1.DOCX]

| **Name** | **Sequence(5'-3')** | **Use** |
| --- | --- | --- |
| invpBac1 | GCGGCGACTGAGATGTCCTAAATG | Inverse PCR to amplify 5’pBac end |
| invpBac2 | GCTTGTTGGTGAGGATTCTGACAGTG |  |
| invpBac3 | GCTTCTAAACGCTTACGCATAAACGATG | Inverse PCR to amplify 3’pBac end |
| invpBac4 | CGATATACAGACCGATAAAACACATGCG |  |
| invpBac1nest | GACGGATTCGCGCTATTTAGAAAGAG | Nested inverse PCR to amplify 5’pBac end (lines #8, #36, #40, #70 and #75) |
| invpBac2nest | AATCTTGACCTTGCCACAGAGGACT |  |
| invpBac3nest | CCATAGGCCACCTATTCGTCTTCCT | Nested inverse PCR to amplify 3’pBac end (lines #8, #36, #40, #70 and #75) |
| invpBac4nest | CGCATGATTATCTTTAACGTACGTCAC |  |
| pBac3'FL_F2 | GTGCCAAAGTTGTTTCTGACTG | PCR to check FL19 insertion of original and new lines |
| pBac3'FL_R2 | CTTCCCATCAAGGATGTGGTAT |  |
| FL19 Sf205_R1 | CTGCATTACATGGTACTGTGTTGA | PCR to check FL19 insertion and homozygosity of line#8 |
| FL19 Sf722_F2 | GCTTTTATAAGGCTTTCCCAGTATC |  |
| FL19 Sf722_R1 | CGATACCCTAAGCACGTTAAAGAT |  |
| X-7.FL19_Scaf10F1 | ACTCACCTCTTTCTTGCTGTCCT | PCR to check FL19 insertion and homozygosity of line# 7(X)+FL19 |
| X-7.FL19_Scaf10R1 | CACTGTTTTTCTATACTGCACTGCT |  |
| Scaf722New_70F | GGGTATGGACGAAATGCTTAGTA | PCR to confirm FL19 location of line#70 |
| Scaf205New_75F | AACTTATGAGGTGCAACTGGAAC | PCR to confirm FL19 location of line#75 |

**Table S1. Oligonucleotide primers used in this study.**
